# DIDS (4,4-Diisothiocyanatostilbenedisulphonic Acid) Induces Apoptotic Cell Death in a Hippocampal Neuronal Cell Line and Is Not Neuroprotective against Ischemic Stress

**DOI:** 10.1371/journal.pone.0060804

**Published:** 2013-04-05

**Authors:** Matthew E. Pamenter, Guy A. Perkins, Xiang Q. Gu, Mark H. Ellisman, Gabriel G. Haddad

**Affiliations:** 1 Department of Pediatrics (Division of Respiratory Medicine), University of California San Diego, La Jolla, California, United States of America; 2 National Center for Microscopy and Imaging Research, University of California San Diego, La Jolla, California, United States of America; 3 Center for Research in Biological Systems, University of California San Diego, La Jolla, California, United States of America; 4 Department of Neuroscience, University of California San Diego, La Jolla, California, United States of America; 5 The Rady Children's Hospital-San Diego, San Diego, California, United States of America; University of Iowa, United States of America

## Abstract

DIDS is a commonly used anion channel antagonist that is putatively cytoprotective against ischemic insult. However, recent reports indicate potentially deleterious secondary effects of DIDS. To assess the impact of DIDS on cellular viability comprehensively we examined neuronal morphology and function through 24 hours treatment with ACSF ± DIDS (40 or 400 µM). Control cells were unchanged, whereas DIDS induced an apoptotic phenotype (chromatin condensation, nuclear fragmentation and cleavage of the nuclear membrane protein lamin A, expression of pro-apoptotic proteins c-Jun N-terminal kinase 3, caspase 3, and cytochrome C, Annexin V staining, RNA degradation, and oligonucleosomal DNA cleavage). These deleterious effects were mediated by DIDS in a dose- and time-dependant manner, such that higher [DIDS] induced apoptosis more rapidly while apoptosis was observed at lower [DIDS] with prolonged exposure. In an apparent paradox, despite a clear overall apoptotic phenotype, certain hallmarks of apoptosis were not present in DIDS treated cells, including mitochondrial fission and loss of plasma membrane integrity. We conclude that DIDS induces apoptosis in cultured hippocampal neurons, in spite of the fact that some common hallmarks of cell death pathways are prevented. These contradictory effects may cause false-positive results in certain assays and future evaluations of DIDS as a neuroprotective agent should incorporate multiple viability assays.

## Introduction

Research to find safe and effective cytoprotective agents against ischemic insult is of pressing clinical need [1]. 4,4-diisothiocyanatostilbenedisulphonic acid, or DIDS, is a commonly-used non-specific anion channel inhibitor that has shown remarkable promise as a cytoprotective agent against ischemic insults in heart and brain, ameliorating up to 90% of cell death in some assays [2], [3], [4], [5], [6]. In addition to ischemia there is also evidence that DIDS is protective against other pathologies, including: beta-amyloid formation in cortical neurons; arsenic-, staurosporine- and ethanol-induced apoptosis in leukemia cells, cortical neurons, and cardiomyocytes, respectively; and volume-dependent apoptosis in cardiomyocytes, epithelial cells, and neurons [7], [8], [9], [10]. The protective effect of DIDS is usually attributed to blockade of anion channels or anion exchangers and associated reductions of Cl^-^ or reactive oxygen species (ROS) flux that regulate cell volume, or downstream stress pathways mediated by toll-like receptors, mitogen-activated protein kinases, protein kinase C, phosphatidylinositol 3-kinase (PI3K)/Akt, or matrix metalloproteinases [2], [4], [5], [6], [8], [11], [12].

Although these results suggest a promising role for DIDS as a cytoprotective agent, several recent studies have suggested that DIDS may have additional effects that are detrimental to the long-term viability of cells. DIDS prevents protein translocation across the ER membrane, uncouples mitochondrial respiration, and directly binds to the DNA binding site of RAD51, an essential eukaryotic DNA recombinase, competitively inhibiting DNA-RAD51 binding activity and double stranded DNA repair [13], [14], [15]. Furthermore, DIDS affects the regulation of ionic and neurotransmitter homeostasis: DIDS reduces tonic astrocyte glutamate release and synaptic ATP uptake in brain, inhibits post-synaptic γ-amino butyric acid (GABA)_A_ receptors, inhibits renal Na^+^/K^+^-ATPase and vesicular H^+^-ATPase activity, and reduces mitochondrial V_m_ and Ca^2+^ buffering in bacterial cells [15], [16], [17], [18], [19], [20]. DIDS also inhibits cellular proliferation in some cell types [15], [21], induces apoptosis in tumors and stem cells [21], [22], and perhaps more importantly, increases cell death in some disease models of pathology. For example, DIDS enhances: vacuole formation and cell death in serum-starved kidney epithelial cells [23], apoptosis in ischemic coronary endothelial cells [24], and lesion expansion in damaged brain by inhibiting microglia process extension [25].

Perhaps most confounding, DIDS may have paradoxical opposing effects on indicators of cell viability depending on the experimental assay utilized. In recent studies we found that DIDS prevents deleterious ROS release and propidium iodide (PI) uptake in ischemic hippocampal neuronal cultures, but depletes neuronal ATP supplies in normoxic control experiments [4], [6]. Taken together, these data point to a myriad of DIDS-mediated interactions that may be simultaneously protective and deleterious to the long-term viability of cells. Therefore, closer examination of the effects of DIDS on cellular viability is critical before DIDS is further considered as a potential neuroprotective agent. To better understand the comprehensive impact of DIDS on cellular viability we examined the effect of DIDS (40 or 400 µM) on cultured hippocampal neurons and assayed neuronal morphology with transmission electron microscopy (TEM), and cellular viability and function by examining [ATP], PI uptake, apoptotic Annexin V staining and the expression of apoptosis-related proteins, mitochondrial morphology and viability, nucleus integrity, and DNA and RNA integrity. We also assessed the ability of DIDS to protect neurons against an *in vitro* mimic of the ischemic penumbra (ischemic solution: IS) using multiple viability assays to carefully evaluate its efficacy as a neuroprotective agent.

## Materials and Methods

### Cell cultures

HT22 mouse hippocampal neurons (a gift from Dr. Pam Maher, Salk Institute, La Jolla, CA [26]) were cultured in Dulbecco's Modified Eagle Medium (DMEM, ATCC) supplemented with 10% bovine calf serum (Hyclone, Santa Clara, CA) and 100 U/ml penicillin/streptomycin (Invitrogen, Carlsbad, CA), and grown at 37°C in a 5% CO_2_ incubator. Cells were grown for 5–8 passages and split when they reached 60–80% confluence. For experiments, cells were seeded into 96-well microplates (Corning, Lowell, MA), glass-bottom 35 mm culture dishes (MatTek, Ashland, MA), or cell culture flasks (Corning) at a density such that when grown overnight they reached ∼70% confluence for experimentation. Samples were treated as specified in the *experimental design* section (below). To reduce sheer stress, cells seeded into multi-well microplates were gently washed with a TECAN PW96/384 Washer (TECAN, San Jose, CA) and then examined visually to ensure cells had not been washed away.

### Experimental design

Neuronal cultures were treated in artificial cerebral spinal fluid (ACSF, in mM: NaCl 129, KCl 5, CaCl_2_ 1.3, MgCl_2_ 1.5, NaHCO_3_ 21, glucose 10, [315 mOsM, pH 7.4, 21% O_2_, 5% CO_2_, balance N_2_]), or in ACSF with DIDS (40 or 400 µM, dissolved in DMSO to a final bath [DMSO]<0.4%), or ACSF with 0.4% DMSO alone (sham vehicle) for up to 24 hrs. In an additional set of experiments, neuronal cultures were treated with an ischemic penumbral perfusate mimic (Ischemic solution: IS, in mM: K^+^ 64, Na^+^ 51, Cl^−^ 77.5, Ca^2+^ 0.13, Mg^2+^ 1.5, glucose 3.0, glutamate 0.1, [315 mOsM, pH 6.5, 1.5% O_2_, 15% CO_2_, balance N_2_]) [4], [27], [28], [29] alone, or in combination with DIDS. All treatments were run simultaneously and in parallel for each assay. Following treatment, samples were assayed as indicated below. Chemicals were purchased from Sigma unless otherwise indicated (Sigma-Aldrich, St. Louis, MO). Solutions were made fresh daily.

### Annexin V assay and confocal microscopy

Annexin V expression was measured following 2 and 24 hrs of treatment using Annexin V-FITC Apoptosis Detection Kits (Enzo Life Sciences, Plymouth Meeting, PA) as per the manufactures instructions. Unless otherwise indicated, microscopy samples were fixed by incubation for 15 mins with 4% paraformaldehyde at room temperature. Samples were washed in PBS and preserved with Prolong Gold antifade reagent with DAPI (Invitrogen) and were stored in the dark at 4°C and imaged within one week. Fixed samples were imaged using 10, 20, and 40× objectives and an Olympus FV1000 scanning confocal microscope using 488 nm (FITC), and 405 nm (DAPI) laser lines (Olympus, San Diego, CA). For data collection, the parameters of the microscope such as light intensity, exposure time, camera gain, etc., were determined for the brightest fluorescing sample and standardized for subsequent samples. Experiments were repeated at least 4 times and for co-localization analysis five random sections from each study group were taken using AxioVision (Carl Zeiss, Thornwood, NY), and the percentage of neurons staining positive for Annexin V was determined by the ratio of FITC-stained cells to DAPI-stained nuclei. Metamorph (Molecular Devices, Sunnyvale, CA) image analysis software was used to count fluorophore-positive stained cells/DAPI-positive cells. Total Annexin V fluorescence was assessed in 96-well microplates (Corning) seeded at a density of ∼50,000 cells per well. Samples were treated for 2 or 24 hrs and then analyzed on a Bio-Tek PowerWave 340 microplate spectrophotometer (Bio-Tek, Winooski, VT, Ex/Em: 485/530 nm) using Gen 5 software (Bio-Tek) within one hour of staining.

### ATP luciferase assay

Total ATP content ([ATP]) was assessed in solid-bottom, black 96-well microplates (Corning) using PerkinElmer ATPlite Luminescence Assay System kits as specified by the manufacturers protocol (PerkinElmer, MA, USA) and measured on a spectrophotometer (Bio-Tek). An equal number of cells (∼50,000) were seeded into each well and [ATP] was assessed following 24 hrs of treatment. Standard curves were generated using serial dilutions of a known ATP standard provided in each kit. The sensitivity of the detector was calibrated to the luminescence of the highest [ATP] standard in each experiment. Results were normalized to ATP luminescence recorded from control cells assayed at *t* = 0 hours. Microplate ATP luciferase experiments were repeated 5 times and each plate contained at least 16 replicate wells of each treatment group. Blank wells and cell-free wells containing each treatment perfusate were also included on each plate, and the final data is corrected for these factors. DIDS quenched luminescence in a dose-dependent fashion and this effect was quantified in a separate experiment by adding serial dilutions of DIDS to serial dilutions of the ATP standard and subtracting the resulting luminescence from ATP standard luminescence measurements in the absence of DIDS on the same plate. Results were corrected for this factor.

### Lamin A cleavage immunohistochemical assay

Samples grown in glass-bottom culture dishes (MatTek) were treated for 24 hrs and then fixed as per the *Annexin V* assay, above. Antigen retrieval was performed in 10 mM citrate buffer (pH 6.0) for 10 mins at 70°C, and then samples were blocked in 10% normal goat serum in PBS (blocking buffer) with 0.3% triton x-100 (0.3% TBST) for 30 mins. Following blocking, samples were incubated in primary antibody (cleaved lamin A, 1∶100; Cell Signaling, Danvers, MA) overnight at 4°C, and then rinsed three times in 0.1 %TBST for 10 mins each. Samples were then incubated in the secondary antibody (anti-rabbit FITC/AlexaFluor-conjugated; 1∶100; Invitrogen) diluted in ½ blocking buffer, for 60 mins at room temperature; then rinsed 3 times in TBST (10 mins each), once in PBS without TX-100, and finally preserved with Prolong Gold antifade reagent with DAPI (Invitrogen), stored in the dark at 4°C, and imaged as per the *Annexin V* assay (above) within one week. Experiments were repeated 4 times.

### Propidium iodide (PI) exclusion assay

PI exclusion was assessed in microplates by incubating samples in 5 µg/ml PI during 24 hrs of treatment and PI fluorescence was immediately assessed on the plate spectrophometer (Ex/Em: 485/630 nm). Microplate PI experiments were repeated 4 times and each plate contained 16 replicate wells each of treatment group. Blank wells and cell-free wells containing each treatment perfusate with PI were also included on each plate, and the final data is corrected for these factors.

### Protein extraction and Western blots

Samples grown in 150 cm^2^ culture flasks were treated for 6 hrs to match similar experiments conducted in our laboratory previously [29]. Following treatment, samples were rinsed twice with PBS and detached from the matrix with a cell scrapper into ice-cold PBS. The resulting cell suspensions were centrifuged at 250×*g* for 5 mins at 4°C, the supernatant was aspirated away, and cells were re-suspended in cell lysis buffer. Samples were then homogenized by vortexing for 60 seconds and proteins were extracted by incubation in lysis buffer with mixing at 4°C for 45 mins, followed by centrifugation for 10 mins at 14,000×*g* at 4°C. Supernatants were taken as whole cell lysates and protein concentration was measured using a bicinchoninic acid kit, according to the manufacturer's instructions (Sigma).

For Western blot analysis, equal amounts of protein (40 µg/well) were separated on 4–12% precast NuPAGE bis-Tris SDS-PAGE gels (Invitrogen) and transferred to polyvinylidene difluoride membranes (Immobilin-P; Millipore, Bedford, MA). Western blots were performed with antibodies against α-actin and cleaved caspase 3 (1∶1,000, Cell Signaling, Danvers, MA); and cytochrome C and JNK3 (1∶200, Santa Cruz Biotechnology, Santa Cruz, CA). Specific bands were visualized after incubation with the respective secondary antibodies (1∶2000) using enhanced chemiluminescense (GE Healthcare/Amersham Biosciences, Buckinghamshire, UK). Following chemiluminescent detection, membranes were stripped of antibodies by incubation in 50 ml of TRIS stripping buffer with 2% SDS and 0.7% β-mercaptoethanol (pH 6.8) for 30 minutes at 50°C, and then subsequently re-probed for other proteins. This process was repeated such that each protein was assayed once per membrane, allowing for direct comparison of protein changes on the same blot. Densitometry of Western blots from each experimental group were obtained (*n* = 3 gels for each), and absolute values were normalized to α-actin expression on the same blot. Results were analyzed in arbitrary units, comparing each value with that obtained from each respective α-actin measurement on each blot, and results are expressed as fold-change relative to untreated controls run simultaneously.

### RNA extraction and quantification

Samples were grown in cell culture flasks (∼1×10^6^ cells per flask) and treated for 1–24 hrs as indicated. Cells were rinsed and detached in ice-cold PBS and then immediately centrifuged with an Eppendorf 5810 R centrifuge at 4°C (Eppendorf, San Diego, CA). The resulting supernatant was discarded and the cell pellet were re-suspended in cell lysis buffer with 1% β-mercaptoethanol and lysed by vortexing. Total RNA was extracted using a Clontech Nucleospin RNA II kit with on-column DNAase step (Macherey-Nagel, Bethlehem, PA). RNA integrity number (RIN) and total RNA (RNA_tot_) were assessed with an Agilent 2100 Bioanalyzer and RNA chips (Agilent, Santa Clara, CA). RIN is the output of an algorithm that assesses RNA integrity, where level 10 RIN is completely intact RNA [30]. Fold-changes in RNA content were determined relative to total RNA in DIDS-free ACSF controls treated simultaneously, and experiments were repeated 4 times.

### Transmission electron microscopy

Cells were grown on 35 mm #0 thickness glass bottom culture dishes (MatTek). Following 24 hrs treatment, samples were fixed with a 37°C solution of 2% paraformaldehyde, 2.5% glutaraldehyde (Ted Pella, Redding, CA) in 0.1 M sodium cacodylate (pH 7.4), and transferred to room temperature for 10 mins, and then incubated for an additional 30 mins on ice. Fixed cultures were rinsed 3 times for 3 mins each with 0.1 M sodium cacodylate plus 3 mM CaCl_2_ (pH 7.4) on ice and then post-fixed with 1% osmium tetroxide (Ted Pella), 0.8% potassium ferrocyanide, and 3 mM CaCl_2_ in 0.1 M sodium cacodylate (pH 7.4) for 60 mins, and were then washed 3 times for 3 mins with ice-cold distilled water. Cultures were finally stained overnight with 2% uranyl acetate at 4°C, dehydrated in graded ethanol baths, and embedded in Durcupan resin (Fluka, St. Louis, MO). Ultrathin (70 nm) sections were post-stained with uranyl acetate and lead salts, and evaluated by a JEOL 1200FX transmission electron microscopy operated at 80 kV. Images were recorded on film at 6,000× magnification. The negatives were digitized at 1,800 dpi using a Nikon Cool scan system, giving an image size of 4033—6010 pixel array and a pixel resolution of 2.35 nm [31]. Images of 20 cells or organelles were taken from each experimental condition. All TEM experiments were repeated at least twice. TEM analysis of mitochondrial, nuclear, and chromatin morphology was performed using standard stereology and other morphometric techniques by measuring at >10 randomly chosen locations from each experimental replicate.

### TUNEL assay

We used an *in situ* fluorescein terminal transferase dUTP nick end-labeling (TUNEL) assay to label apoptotic DNA strand breaks (Roche, Mannheim, Germany). Following 24 hrs treatment, samples were fixed and processed as per the manufactures instructions for adherent cells with modifications to enhance TUNEL permeability. Briefly, samples were fixed in 2% paraformaldehyde for 30 mins at room temperature, rinsed in PBS and then permeabilised with 0.1% Triton X-100 with 1% sodium citrate for 15 mins at room temperature, and then treated for 0.1 M sodium citrate at 70°C for 30 mins. Samples were rinsed in PBS, and then incubated in the TUNEL reaction mixture for 60 mins in the dark at 37°C and then rinsed with PBS and preserved. Samples were imaged using the same confocal microcopy protocol as the *Annexin V assay* (above).

### Statistics

Data were analyzed using a two-tailed Student *t*-test or one-way analysis of variance (ANOVA), followed by Dunnet's post-test. Significances were indicated if *P*<0.05 assuming two groups had an equal variance. Statistical analysis was performed using Prism software (GraphPad, San Diego, CA).

## Results

### DIDS induces an apoptotic phenotype but plasma membrane integrity is preserved

**Figure 1:**
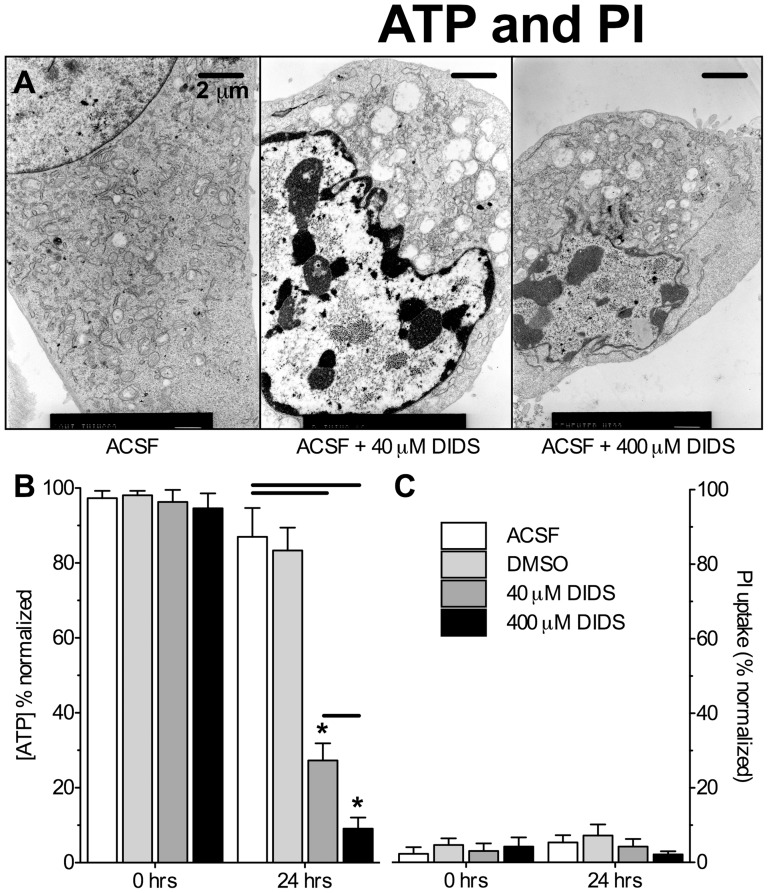
DIDS treated cells retain plasma membrane integrity despite ATP depletion and an apoptotic phenotype. DIDS treatment induced an apoptotic phenotype and also dose-dependant [ATP] depletion from neurons, but membrane integrity was preserved. (**A**) Sample TEM images of neurons treated as indicated for 24 hrs. TEM experiments were repeated 2 times and 10–20 cells were examined from each treatment group. (**B**) Summary of neuronal [ATP] versus [DIDS] (40 or 400 µM) following 24 hrs treatment, normalized to untreated controls at *t* = 0. ATP luciferase experiments were repeated 5 times. (**C**) Summary of neuronal propidium iodide (PI) uptake vs. [DIDS] following 24 hrs of treatment, normalized to untreated controls. PI exclusion experiments were repeated 4 times. [DMSO] = 0.4%. Data are mean ± SEM. Asterisks (*) indicate significant difference from normoxic controls; bars indicate significance between treatments (*p*<0.05).

We have previously reported that DIDS treatment (400 µM) prevents plasma membrane degradation during an ischemic insult as evidenced by the absence of propidium iodide (PI) uptake or lactate dehydrogenase release [5], [6], [32]. On the other hand, DIDS depletes neuronal [ATP] following 24 hrs of treatment in neuronal cell lines [4]. Therefore, we first examined the effect of DIDS at both the concentration we have previously reported to be neuroprotective and also at 10-fold lower concentrations (40 or 400 µM) on hippocampal neuronal morphology using TEM analysis, and also assayed [ATP] and plasma membrane integrity (via PI exclusion). Control cells treated in ACSF alone or with ACSF plus 0.4% DMSO (vehicle sham) for 24 hrs had a normal morphology, with intact plasma membranes, large nuclei free of apoptotic chromatin condensation, and healthy-appearing organelles (Fig. 1A, TEM images are representative of 20 images from 2 experiments per treatment). Furthermore, [ATP] was not significantly depleted in ACSF treated neurons at 24 hrs relative to untreated controls at *t* = 0 (Fig. 1B, *n* = 16 replicates from 5 individual experiments each) and plasma membranes excluded PI (Fig. 1C, *n* = 16 replicates from 4 individual experiments each; note: for positive controls of PI uptake in this cell line please see Fig. 1 of reference [6]).

Neurons treated with DIDS for 24 hrs had an apoptotic phenotype (Fig. 1A) [33], [34]. DIDS treatment also caused a dose-dependant depletion of neuronal [ATP], and following 24 hrs treatment neurons treated with 40 µM or 400 µM DIDS retained 27.3±4.6 and 9.1±3.0% of their [ATP], respectively, relative to untreated controls (Fig. 1B). Interestingly, DIDS-treated neurons did not take up PI (Fig. 1C), indicating that plasma membrane degradation, which is typical of both apoptotic and necrotic cell death [33], was prevented by DIDS treatment despite the appearance of a putatively apoptotic phenotype and extensive ATP depletion.

### DIDS induces translocation of the apoptotic marker Annexin V in a dose- and time-dependant manner

**Figure 2:**
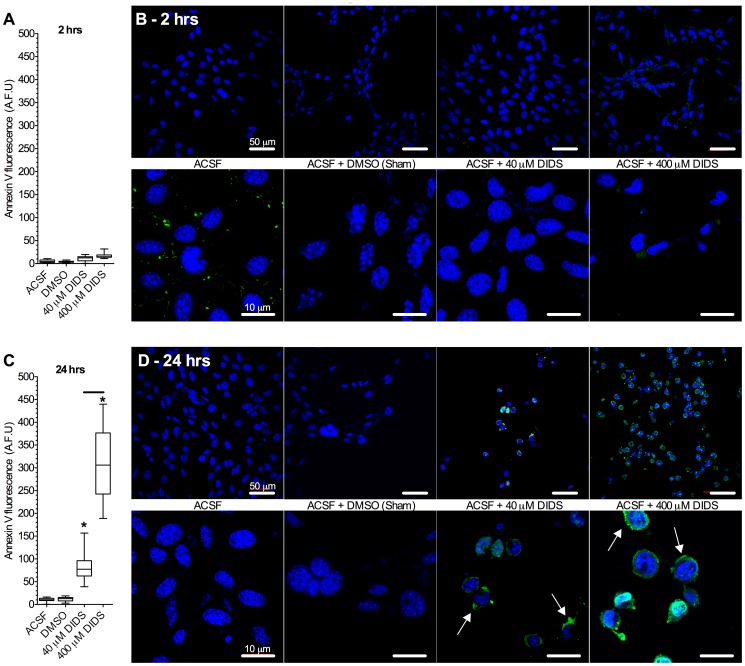
DIDS induces Annexin V staining in a dose- and time-dependant manner. (**A**) Summary of Annexin V fluorescence from neurons treated for 2 hrs in 96-well microplates as indicated. (**B**) Sample confocal microscopy images of Annexin V (green) and DAPI (blue) fluorescence from neurons treated as indicated for 2 hrs. (**C**) Summary of Annexin V fluorescence from neurons treated for 24 hrs in 96-well microplates as indicated. (**D**) Sample confocal microscopy images of Annexin V (green, and arrows) and DAPI (blue) fluorescence from neurons treated as indicated for 24 hrs. Images are representative of 4 separate experiments. Data are mean ± SEM. Asterisks (*) indicated significant difference from normoxic controls; bars indicate significance between treatments (*p*<0.05). AFU = artificial fluorescence units.

To determine whether DIDS activated apoptotic pathways in neurons we next examined the apoptotic translocation of phosphatidylserine residues that bind Annexin V to the extracellular plasma membrane surface following 2 and 24 hrs of treatment with ACSF±DMSO±DIDS [35], [36]. In control cells (treated with ACSF alone or with ACSF plus DMSO (vehicle sham)) Annexin V fluorescence was not apparent at either time point in either fluorescent microplate assays or confocal microscopic imaging (Figs. 2A–D, *n* = 16 replicates from 4 experiments each for microplate assays and 4 experiments for confocal imaging assays). Conversely, DIDS induced Annexin V binding in a dose- and time-dependant fashion. At 2 hrs extensive Annexin V fluorescence was not observed (Fig. 2A&B); whereas following 24 hrs treatment, strong Annexin V fluorescence was detected at all [DIDS] and samples treated with 400 µM DIDS exhibited ∼3-fold greater Annexin V fluorescence than samples treated with 40 µM DIDS.

### DIDS upregulates apoptotic pathways

**Figure 3:**
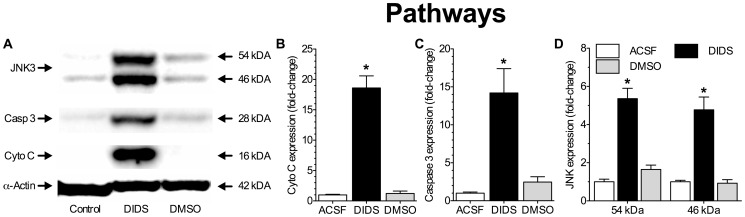
DIDS upregulates apoptotic pathways in neurons. (**A**) Sample Western blots of apoptosis-related protein expression from neurons treated as indicated for 6 hrs. (**B–D**) Summaries of fold-change in cytochrome C (**B**), caspase 3 (**C**), and JNK3 (**D**) protein expressions from (A) normalized to α–actin expression in the same sample. Data are mean ± SEM from 3 separate experiments for each protein and treatment. Asterisks (*) indicate significant difference from untreated controls (*p*<0.05).

To better understand the cell death pathways initiated by DIDS-treatment we next examined changes in the expression of proteins related to cellular apoptosis. Apoptosis, mediated either by cytochrome C release from ruptured mitochondria or by immune receptor-mediated c-Jun N-terminal kinase 3 (JNK3) activation induces caspase-dependant cascades; which are molecularly characterized by activation of caspase 3, among other caspases [37], [38]. In ACSF or DMSO-alone control samples, cytochrome C, caspase 3 and JNK3 proteins were not expressed (*n* = 3 for each treatment, Fig. 3A–D). In contrast, DIDS-treated cells had a molecular profile typical of apoptosis, including increases of ∼18-fold in cytochrome c expression (Fig. 3A&B); ∼14-fold in caspase 3 expression (Fig. 3A&C); and ∼5-fold in JNK3 expression (Fig. 3A&D).

### Mitochondria and nuclei in DIDS treated neurons exhibit phenotypes typical of cell death

**Figure 4:**
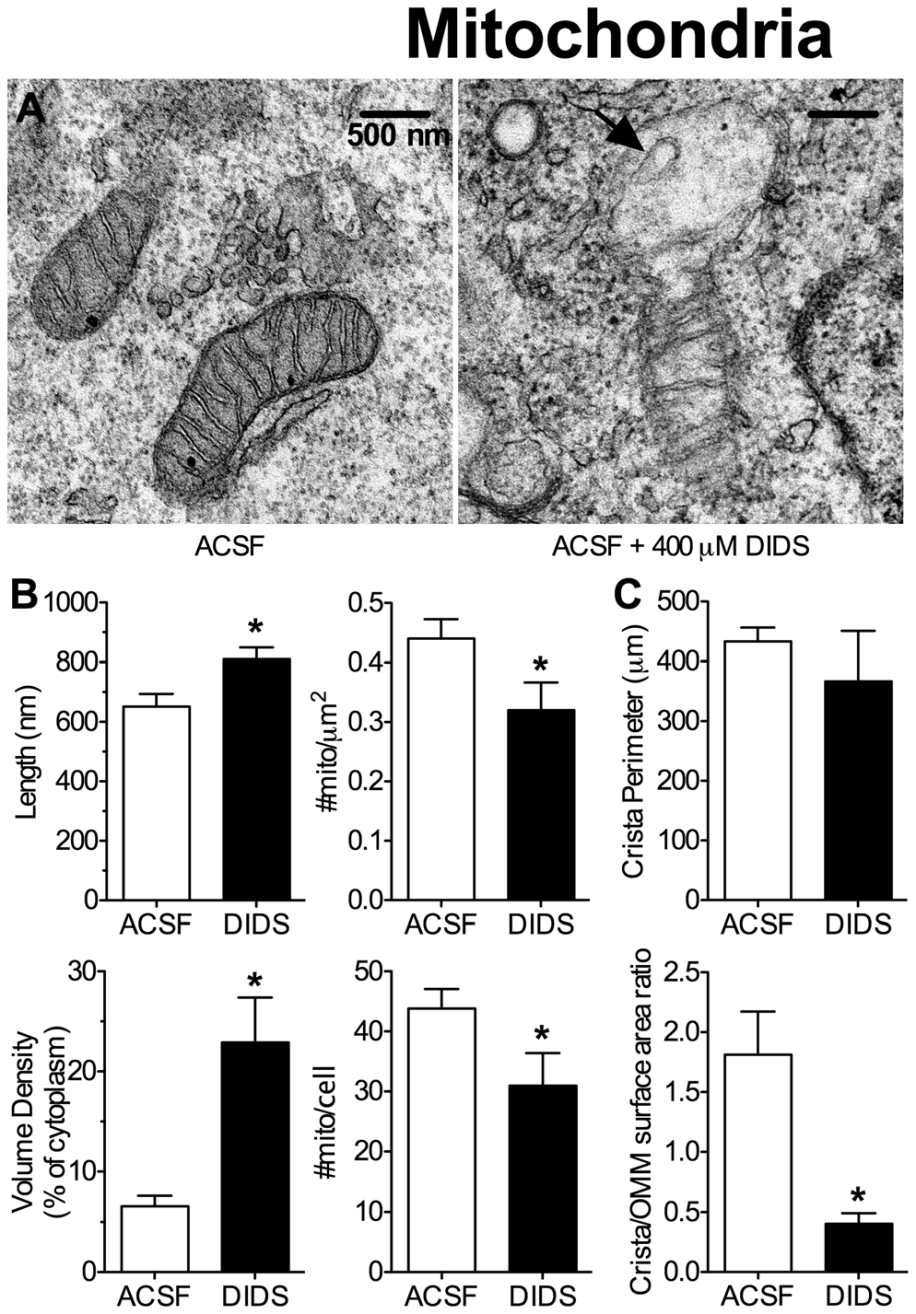
DIDS (400 µM) causes mitochondrial swelling and cristae reduction. DIDS treated mitochondria were longer, fewer in number, and had markedly reduced cristae density. (**A**) Sample TEM images of mitochondria from neurons treated as indicated for 24 hrs. Arrow indicates “ballooning” of outer mitochondrial membrane (OMM). (**B**) Summary of mitochondrial morphology-related parameters from Fig. 4A. (**C**) Summary of cristae morphology-related parameters from Fig. 4A. Data are mean ± SEM. Asterisks (*) indicate significant difference from normoxic controls (*p*<0.05).

Mitochondria and nuclei are key components of cell death pathways [33], [39]. Therefore, we next examined the effect of DIDS treatment (400 µM) on mitochondrial and nuclear morphology using TEM analysis and stereology techniques. Mitochondria in cells treated with DIDS for 24 hrs were longer, swollen, and less numerous relative to control mitochondria (treated in ACSF) (Fig. 4A&B, *n* = 20 cells from 2 experiments per treatment). In addition, mitochondrial membranes were distended and balloon-like in appearance (Fig. 4A, arrow); and cristae networks were irregular, with reduced cristae density relative to mitochondrial membrane surface area (Fig. 4C).

**Figure 5:**
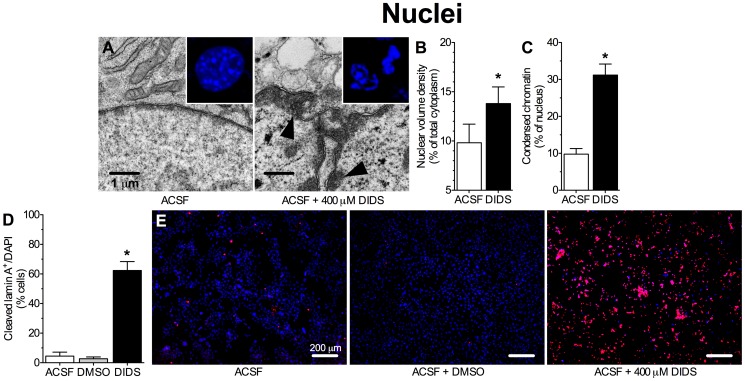
DIDS (400 µM) treated nuclei exhibit apoptotic chromatin condensation, nuclear envelope shrinkage and fragmentation of the nuclear membrane into apoptotic bodies. (**A**) Sample TEM images of nuclei from neurons treated as indicated for 24 hrs. Arrows indicate condensed chromatin beads. Inset: DAPI-stained chromatin distribution in healthy (ACSF) and apoptotic (DIDS-treated) nuclei. (**B**) Summary of nuclear volume density from samples in (A). (**C**) Summary of chromatin volume density from samples in (A). (**D**) Summary of cleaved lamin A expression normalized to DAPI-stained nuclei following 24 hrs treatment. (**E**) Sample immunohistochemical (IHC) fluorescent confocal microscopy images of cleaved lamin A (red) and DAPI (blue) staining in neurons from Fig. 5D. Lamin A IHC experiments were repeated 4 times. Data are mean ± SEM. Asterisks (*) indicate significant difference from normoxic controls (*p*<0.05).

Apoptotic changes to nuclear morphology and chromatin distribution are well characterized and include chromatin condensation and nuclear membrane distortion and fragmentation into discrete apoptotic bodies [33], [34], [40]. In control cells, nuclei were smooth and oval in shape, and chromatin was distributed primarily in nucleosomes inside nuclei (Fig. 1A and inset). Conversely, nuclei in cells treated with DIDS for 24 hrs were distorted and swollen, taking up a greater percentage of the cytoplasm relative to controls (Fig. 5A&B). Chromatin was densely aggregated at the nuclear membrane (chromatin condensation increased from 9.8±1.5% to 31.2±3.0%), which was in turn fragmented into apoptotic bodies in DIDS treated neurons (Fig. 5A–C, *n* = 20 nuclei from 2 experiments per treatment). We have previously demonstrated that DIDS prevents pathology- or proteinase-mediated plasma membrane degradation in cultured neurons by inhibiting the function of cellular enzymes responsible for cleavage of plasma membrane components [6]. To determine whether this effect extends to intracellular digestion of nuclear membrane components, we examined the effect of DIDS on lamin A cleavage, since lamin A is an integral component of nuclear membranes whose cleavage is typical of apoptosis in these cells [40] and whose activity is required for nuclear membrane fragmentation. In good agreement with our observation that DIDS treated nuclei appeared fragmented in TEM and DAPI-examination (Fig. 5A and inset), cleaved lamin A expression increased with DIDS treatment and the percentage of cells expressing cleaved lamin A increased from 4.5±2.7% in control cells, to 62.4±6.3% in DIDS-treated samples (Fig. 5D&E, *n* = 4 experiments).

**Figure 6:**
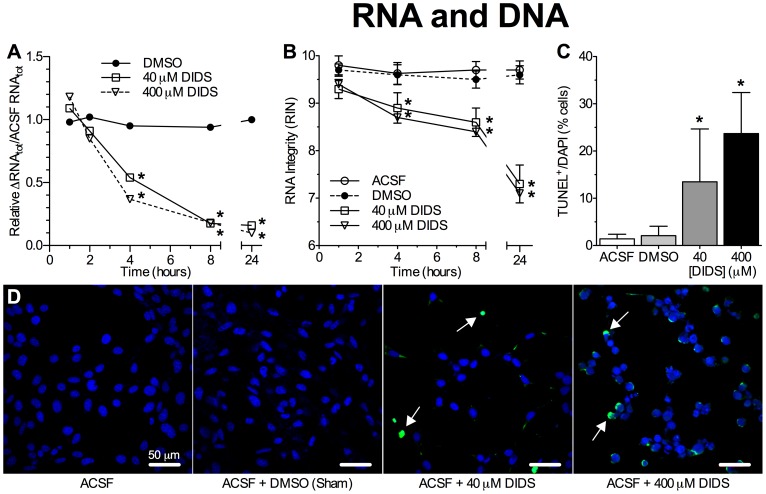
DIDS causes RNA and DNA degradation in a dose-dependant manner. (**A&B**) Summaries of the effect of ACSF±DMSO±DIDS (40 or 400 µm) on total RNA content (RNA_tot_) (A) or RNA integrity (RIN) (B) from neurons treated for 1, 2, 4, 8 or 24 hrs as indicated. RNA_tot_ was normalized to untreated samples. RNA experiments were repeated 4 times at each time point. (**C**) Summary of the ratio of terminal transferase dUTP nick end-labeling (TUNEL)-positive neurons relative to DAPI-stained nuclei from Fig. 6D. (**D**) Sample confocal microscopy images of TUNEL (green, arrows) and DAPI (blue) fluorescence from neurons treated as indicated for 24 hrs. TUNEL experiments were repeated 4 times. Data are mean ± SEM. Asterisks (*) indicate significant difference from normoxic controls; bars indicate significance between treatments (*p*<0.05).

Finally, since DNA cleavage and RNA degradation are also typical of apoptosis [33], [41], [42] and since DIDS has been shown to inhibit cellular DNA repair mechanisms [13], we next examined dose-dependant effect of DIDS treatment on RNA and DNA integrity. In this analysis 40 or 400 µM DIDS caused rapid reductions in the quantity and quality of neuronal RNA, such that following 4 hrs treatment total RNA (RNA_tot_) was reduced by ∼50% in both treatment groups, while the RNA integrity number (RIN) dropped from >9.7 in controls, to <9 (Fig. 6A&B, *n* = 4 for each time point). Following 8 hrs treatment, RNA_tot_ decreased to <20% of ACSF or ACSF plus DMSO-treated controls at the same time point, while RIN decreased to ∼8.5, and RNA quality degraded further to ∼7.0 (RIN) following 24 hrs of treatment. Similarly, DIDS induced DNA degradation in a dose-dependant manner. Following 24 hrs of treatment with 40 µM DIDS 13.5±11.2% of neurons were TUNEL-positive (Fig. 6D, *n* = 4 for each treatment). Conversely, when treated with 400 µM DIDS, 23.7±8.7% of neurons were TUNEL-positive following 24 hrs. ACSF or DMSO-alone did not induce TUNEL staining (Fig. 6D).

### DIDS does not protect against ischemic insult

**Figure 7:**
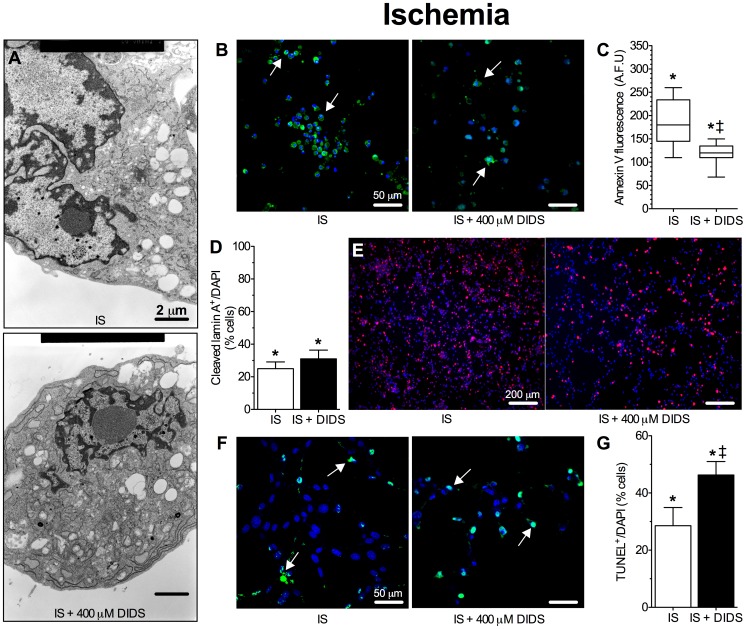
DIDS is not neuroprotective against an *in vitro* ischemic stress. Ischemic solution (IS) treatment induced an apoptotic phenotype in neurons and DIDS was not cytoprotective against this stress. (**A**) Sample TEM images of neurons treated as indicated for 24 hrs. TEM experiments were repeated 2 times and 10–20 cells were examined from each treatment group. (**B**) Sample confocal microscopy images of Annexin V (green, arrows) and DAPI (blue) fluorescence from neurons treated as indicated for 24 hrs. (**C**) Summary of Annexin V fluorescence from neurons treated for 24 hrs in 96-well microplates as indicated. Images are representative of 4 separate experiments. AFU = artificial fluorescence units. (**D**) Summary of cleaved lamin A expression normalized to DAPI-stained nuclei following 24 hrs treatment. (**E**) Sample immunohistochemical (IHC) fluorescent confocal microscopy images of cleaved lamin A (red) and DAPI (blue) staining in neurons from Fig. 7D. Lamin A IHC experiments were repeated 4 times. (**F**) Sample confocal microscopy images of TUNEL (green, arrows) and DAPI (blue) fluorescence from neurons treated as indicated for 24 hrs. (**G**) Summary of the ratio of TUNEL-positive neurons relative to DAPI-stained nuclei from Fig. 7F. TUNEL experiments were repeated 4 times. Data are mean ± SEM. Asterisks (*) indicate significant difference from normoxic controls; double daggers (‡) indicate significance between DIDS-treated and DIDS-free samples (*p*<0.05).

We have previously reported that treatment with our *in vitro* mimic of the ischemic penumbra (ischemic solution: IS) induces an apoptotic phenotype in cultured HT22 hippocampal neurons [4], [5], [6], [27], [28], [29]. Furthermore, we have reported that DIDS prevents plasma membrane degradation but not [ATP] depletion in HT22 cells treated with IS [4], [6]. To resolve this seeming phenotypic paradox and to determine if DIDS is a viable neuroprotective candidate against ischemic stress in these cells, we next comprehensively assessed cellular viability in samples treated with IS±DIDS using a variety of the assays described above. IS-treated and IS+DIDS treated cells had a typical apoptotic morphology [4] (Fig. 7A, TEM images are representative of 20 images from 2 experiments per treatment). Apoptotic Annexin V fluorescence was observed in both treatment groups (Fig. 7B&C, *n* = 16 replicates from 4 experiments each for microplate assays and 4 experiments for confocal imaging assays). DIDS-treatment reduced total Annexin V fluorescence ∼25% relative to IS-alone; however, Annexin V staining remained >6–7-fold higher than ACSF-treated controls (see Fig. 2C). Cleaved lamin A expression increased equally with IS or IS+DIDS treatment and the percentage of cells expressing cleaved lamin A was 25.2±4.1 and 31.8±5.4%, respectively, following 24 hrs treatment (Fig. 5D&E, *n* = 4 experiments). Finally, IS or IS+DIDS each induced DNA degradation in a dose-dependant manner. Following 24 hrs of treatment with IS alone 28.6±6.3% of neurons were TUNEL-positive (Fig. 7F, *n* = 4 for each treatment). Conversely, when neurons were co-treated with IS plus 400 µM DIDS, the percentage of TUNEL-positive cells increased to 46.3±4.7%.

## Discussion

In the present study we report that DIDS induces apoptosis in cultured murine hippocampal neurons in a dose- and time-dependant fashion. This conclusion is supported by our observations that DIDS-treatment: 1) depleted neuronal [ATP], 2) caused Annexin-V binding at the plasma membrane, 3) upregulated the expression of three key apoptosis-related proteins, 4) induced an apoptotic phenotype in nuclei (i.e. chromatin condensation, nuclear envelope fragmentation and cleavage), and 5) degraded DNA and RNA. These morphological, biochemical, and molecular phenotypes are all consistent with the induction of apoptosis [4], [11], [33], [34], [35], [41], [42]. In addition, we utilized several assays to carefully evaluate the neuroprotective efficacy of DIDS against an *in vitro* ischemic insult [4], [5], [6], [27], [28], [29]. In this analysis DIDS failed to prevent Annexin V expression, lamin A cleavage, and TUNEL staining in neurons. Therefore, we conclude that DIDS is not neuroprotective against ischemic insults, even though in previous studies we have reported that DIDS prevents neuronal plasma membrane degradation and ROS generation due to ischemic stress [4], [5], [6], [28].

A key finding of our study is that some hallmarks of apoptosis are prevented by DIDS despite overwhelming evidence of apoptotic cell death in parallel assays. For example, we do not observe mitochondrial fission in DIDS-treated cells, which is typical of apoptotic cell death [43]. Instead, mitochondria in DIDS-treated samples are longer than in controls due to swelling and “ballooning” of the outer membrane. The mechanisms behind this difference are unclear but DIDS is known to block a variety of mitochondrial anion channels and flux of Cl^−^ is central to the osmoregulation of cells and organelles [10], [44]. It is tempting therefore to speculate that in apoptotic cells, DIDS inhibits anion flux, resulting in the observed swelling of mitochondria. This then may inhibit the progression of mitochondrial fission in otherwise apoptotic cells.

Similarly, DIDS quenched luciferase luminescence in [ATP] assays in a dose-dependant fashion (see discussion in methods). We corrected for this complication in our data analysis; however, this effect may confound data analysis if proper controls are not performed. Perhaps the strongest example of how DIDS may confound experimental conclusions was our observation that DIDS prevents propidium iodide uptake into apoptotic cells. This result is particularly relevant to pathological research as many studies rely on membrane integrity assays (e.g. PI uptake, alamar blue or trypan blue exclusion, lactate dehydrogenase leakage) to assess cell viability. Indeed, the degradation of plasma membrane integrity is a commonly-shared hallmark of cell death pathways [33]. In necrotic cell death, rapid osmotic changes lead to membrane rupture, whereas in apoptosis, membranes are actively cleaved by proteinases, in particular matrix metalloproteinases (MMPs) [33], [45]. Previously, our laboratory has reported that DIDS prevents vital dye uptake and also lactate dehydrogenase release from primary mouse hippocampal cultures and from murine cell lines [4], [5], [32]. More recently, we have shown that DIDS prevents the vesicular release and subsequent extracellular activation of MMPs following ischemic insult in cultured murine hippocampal neurons [6]. Through this action, DIDS prevents the programmed dismantling of apoptotic neuronal membranes, vital dyes (PI and trypan blue) are excluded from cells, and membrane blebbing events are abolished. In that report we postulated that this action of DIDS prevented apoptotic membrane rupture and the release of deleterious cytoplasmic contents into the extracellular milieu during ischemic stress. The prevention of such release would abrogate the extracellular accumulation of pro-apoptotic and –immunogenic signaling molecules from damaged cells and also reduce ionic and pH alterations of the local perfusate that would otherwise activate cell death pathways in surrounding cells. In this fashion, DIDS may retard the spread of the infarct core into the ischemic penumbra. Our present study suggests that although DIDS may limit the spread of cell death signals between cells, DIDS itself upregulates cell death, and thus likely cancels out this putative benefit.

Our observations that DIDS induces apoptotic Annexin V staining and increases the expression of common apoptotic proteins cytochrome C, cleaved lamin A, caspase 3, and JNK3 clearly supports our conclusion that DIDS is pro-apoptotic [29], [36], [46], [47]. JNK3 is a mediator of immune-receptor mediated apoptotic pathways, while cytochrome C is released from damaged mitochondria in mitochondria-dependent apoptotic pathways [48], [49], [50], [51], [52]. Both of these mechanisms converge to activate caspases, which regulate the progression of apoptosis, including lamin A cleavage and dismantling of the nuclear envelope, DNA and RNA degradation, plasma membrane cleavage, etc. The increased expression of both JNK3 and cytochrome C indicates that apoptosis is induced via multiple pathways by DIDS. Nonetheless, the explicit mechanisms via which DIDS induces cell death are not clear from our study, and as we discuss in the introduction, DIDS has myriad cellular interactions that may induce apoptosis.

The primary canonical action of DIDS is as a Cl^−^ channel or anion exchanger antagonist such that, as a whole, DIDS impairs Cl^−^ movement across the plasma and organelle membranes of cells [10], [23], [44]. Furthermore, DIDS has been shown to inhibit the activity of GABA_A_ receptors, which are a primary entry point of Cl^−^ at post-synaptic neurons during inhibitory synaptic events [19], [20]. The effect of Cl^−^ movement through neuronal GABA_A_ receptors is dependent upon the expression of the Cl^−^ co-transporters NKCC1 and KCC2 [53]. In particular, NKCC1 expression is high in developing neurons and pumps Cl^−^ into cells, whereas KCC2 expression is high in most mature neurons and pumps Cl^−^ out of the cells. Thus, in developing neurons the opening of Cl^−^ channels leads to excitatory Cl^−^ efflux, while in most mature neurons, activation of Cl^−^ channels typically leads to inhibitory Cl^−^ influx [53]. Inhibition of Cl^−^ flux in adult neurons such as those used in our study would therefore limit the impact of inhibitory synaptic transmission and enhance excitatory electrical activity such as occurs during ischemic insult in brain. In a previous paper we examined the effects of DIDS and also the general Cl^−^ channel antagonist 5-nitro-2-(3-phenylpropylamino)benzoic acid (NPPB) on [ATP] depletion in normoxic and IS-treated HT22 neurons and found that DIDS but not NPPB depleted [ATP] in normoxic neurons, while NPPB but not DIDS preserved [ATP] in IS-treated neurons [4]. These results indicate that although Cl^−^ channel antagonism is neuroprotective during ischemia, the deleterious effect of DIDS on cellular viability is not mediated through Cl^−^ channel modulation. Alternatively, protective effects of DIDS-mediated Cl^−^ channel inhibition are masked by deleterious secondary interactions in our experiments. In either case, it is unlikely that the deleterious effects of DIDS on neuronal viability are mediated by modulation of anion transport. The observation that Cl^−^ channel antagonism is neuroprotective deserves further study; however, our results indicate that such studies would be more wisely performed using more specific Cl^−^ channel antagonists instead of DIDS, which has multiple cellular interactions.

Of these secondary interactions, DIDS may induce cell death in our experiments by uncoupling mitochondrial respiration since ATP levels are sharply depleted by DIDS-treatment [15]. Alternatively, inhibition of ATPases, protein synthesis and translocation, DNA repair, Ca^2+^ homeostasis, or proper neurotransmitter function/responses at both the pre- and post-synaptic cell may all contributed to the induction of apoptosis and spread of cell death by DIDS in our study [13], [14], [15], [16], [17], [18], [20], [54]. For example, in our experiments, DIDS activates the JNK3 immune pathway and mitochondrial cytochrome C release, and activates multiple caspase-dependent pathways, including caspases 3 and 6 (since DIDS induces lamin A cleavage, which is a caspase-6 dependent process that leads to nuclear deregulation and cell death [55], [56], [57], cellular events which are also induced by DIDS in our study), indicating that multiple secondary mechanisms are likely activated by DIDS that contribute to the overall deleterious phenotype.

Despite the overall cytotoxic effects of DIDS treatment on cellular viability, the possibility remains that protective effects mediated by DIDS may mitigate more deleterious stresses due to ischemia and provide an overall positive benefit to stroke-afflicted cells and tissues. Indeed, we have previously reported that DIDS treatment reduces deleterious ROS production from ischemic neurons and also prevents the activation of MMPs that regulate the apoptotic cleavage of plasma membranes from a variety of cell types [4], [6]. Therefore, in our present study we have also comprehensively evaluated the ability of DIDS to protect cells from an ischemic stress. From this analysis, we conclude that DIDS is not cytoprotective against ischemia since neurons treated with both IS alone or IS+DIDS have a similar apoptotic phenotype, express Annexin V and cleaved Lamin A protein, and are TUNEL positive. DIDS does slightly reduce the expression of Annexin V in these cells, but DIDS also increases the prevalence of TUNEL staining. Taken together, these data indicate that individual cells are not protected against ischemic stress by DIDS. However, we cannot dismiss the potential wider benefits of DIDS based on these results. Our previous observation that DIDS prevents MMP activation indicate that DIDS could reduce local cell rupture and impair the release of pro-apoptotic agents from dying infarct core cells. In this manner, DIDS might retard the spread of the ischemic penumbra and provide systemic benefits even if secondary effects of DIDS poisons local cells.

The ability of DIDS to simultaneously induce cell death, while preventing some hallmarks of the upregulated cell death pathway is an important observation and calls into question many previous studies examining the protective efficacy of DIDS against various pathological stresses. DIDS is a “dirty molecule” and has been shown to affect cellular function and activity ranging widely from membrane and organelle protein trafficking and function, to synaptic transmission, to mechanisms of DNA repair [13], [14], [15], [16], [21], [44]. Although some cellular interactions of DIDS may be neuroprotective (e.g. antagonism of deleterious reactive oxygen species or ion movement through organelle and plasma membrane Cl^−^ channels) [2], [3], [4], [10], [32], [44], it is not surprising that these multiple effects, in aggregate, are deleterious to cellular viability. Our present results indicate that DIDS is deleterious to neuronal viability and is therefore not suitable for use as a neuroprotective agent due to its wide-ranging non-specific secondary cellular effects. Furthermore, our results should serve as a cautionary note in the evaluation of such drug candidates, and particularly highlights the importance of using multiple assays in parallel to assess cellular viability in such studies.
